# Public transport accessibility indicators to urban and regional services in Great Britain

**DOI:** 10.1038/s41597-023-02890-w

**Published:** 2024-01-09

**Authors:** J. Rafael Verduzco Torres, David Philip McArthur

**Affiliations:** Urban Big Data Centre, University of Glasgow, Glasgow, United Kingdom

**Keywords:** Geography, Society, Carbon and energy

## Abstract

Public transport accessibility to urban and regional services has been found to relate to various social and economic processes, such as unemployment, transport mode choice, property prices, and public health. A frequent type of measures representing accessibility are location-based. While these offer advantages, like flexibility and ease of interpretation, their estimation usually requires specialized skills and substantial computational resources. To lower these barriers, we have prepared a suite of accessibility indicators for key services across Great Britain at a spatially disaggregated level. The dataset includes ready-to-use public transport accessibility indicators for employment, general practitioners (GP, or family physician), hospitals, grocery stores, supermarkets, primary and secondary schools, and urban centres. It also includes the raw travel time matrix from each origin to every potential destination, a primary input for such indicator estimation. Altogether, this resource offers various levels of application, from direct input into a range of research topics to the foundation for creating comprehensive custom indicators.

## Background & Summary

Accessibility indicators aim to measure “the ease of reaching valued destinations”^1^. Public transport accessibility has been found to influence human behaviour and urban and regional processes such as unemployment rates^2^, mode choice^3^, property prices^4^, public health^5,6^, and social inequity^7^. Accessibility is key for evaluating and addressing equity objectives in transport planning, policy, and projects^8^. In fact, accessibility is a valuable equity indicator^9^. Recent research has illustrated its impact for the evaluation of public transport infrastructure supplementing conventional economic approaches including a social equity component in a variety of contexts, in Europe^8,10^, China^11^, and Australia^12^.

There are several ways in which the accessibility can be operationalised, e.g. infrastructure-based, location-based, person-based, and utility-based. Location-based measures are frequently used in research given their ease of communication, theoretical robustness, and availability of data^13^. These types of measures essentially include the following elements: a location of origin (*i*), weights or opportunities (*W*) of type *k* at potential destinations (*j*), and the travel cost between *i* and *j*, *c*_*ij*_. The travel cost has been suggested to be better represented by the travel time rather than straight-line or road network metric distance^14^. The following equation presents the general specification of location-based measures^15^.

$${A}_{ik}=\mathop{\sum }\limits_{j=1}^{n}\,g\left({W}_{jk}\right)f\left({c}_{ij}\right)$$

Accessibility (*A*) is given as the sum of a product of two functions, one corresponding to opportunities and other representing the travel cost. The latter represents a spatial decay function^14^ and it is usually referred to as impedance.

Computing public transport accessibility measures involves challenges for some researchers outside the transport field, though they may still advance their work by incorporating these measures. Barriers may include a requirement for coding skills, specific technical knowledge of public transport timetable formats, or the use of specialised software. In addition, the estimation at a fine-grained level demands considerable computational resources which are not always available to researchers^16,17^. This is chiefly because they involve the estimation of an ‘all-to-all’ travel time matrix (TTM). Thus, our aim is to generate a set of ready-to-use accessibility indicators for the whole of Great Britain (GB) to a variety of key urban and regional services.

This dataset offers public transport accessibility indicators for the year 2021 at the lower super output area (LSOA) level in England and Wales or data zone (DZ) level in Scotland to the following key services: employment, general practitioners (GP, or family physician), hospitals, grocery stores, supermarkets, primary schools, secondary schools, and urban centres. It also includes the raw all-to-all travel time matrix used as the primary input to estimate the indicators. Altogether, the dataset is designed to be a flexible product and is expected to be useful at the following three levels:

1. Foundational level: here, it can serve as a direct input in performance analyses for transportation or planning agencies or for research in a variety of fields at a small official statistical areal unit.
2. Integrative level: at this intermediate level, users or agencies can aggregate the indices either geographically or thematically according to the local context or research needs or use it as input for the construction of complex or tailored measures.
3. Advanced or strategic level: At this deepest level, researchers, public agencies or data service centres can use this contribution alongside the open-source code as a guide for the update or extension of similar indicators in the British or other international context.

The ‘Usage Notes’ section develops these suggestions.

In comparison to similar previous datasets (e.g. Journey time statistics, Department for Transport, DfT^18^), this covers all regions in GB, all data inputs are open-access, all the software used is open-source, provides the raw travel-time matrix, and the source-code is openly available.

## Methods

This dataset offers two types of location-based measures, namely cumulative and ‘dual′ or minimum travel time. The specific type used for the services included is based on the characteristics of the service. For instance, if a service can be easily replaced or they compete with each other (e.g., employment, groceries), a cumulative measure is more suitable.

The notation for cumulative accessibility measures, *A*, is as following:

$${A}_{ik}=\mathop{\sum }\limits_{j=1}^{n}{W}_{jk}f\left({c}_{ij}\right)$$

$$f\left({c}_{ij}\right)=\left\{\begin{array}{ll}1\,{\rm{if}} & {c}_{ij}\le \bar{t}\left({\rm{threshold}}\,{\rm{value}}\right)\\ 0 & {\rm{otherwise}}.\end{array}\right.$$

Here, *i* represents an origin location for which the indices will be estimated, *j* a potential destination, and *W* the size of a service of type *k*. In the cumulative formulation, the impedance function, *f*(*c*_*ij*_), takes a value binary value, 0 or 1. The $\bar{t}$ parameter represents a threshold value which assumes people are equally happy to consider opportunities within a maximum travel time. Thus, they will consider nothing beyond that threshold. Here, travel costs, *c*_*ij*_, are represented by the estimated travel time, integrating the path and road network with public transport services. This method, chosen for its enhanced realism over ‘beeline’ or simple road network distance, incorporates operational transport service characteristics (e.g., frequencies, overlaps, time availability), network structure, and built environment attributes. Further details are provided in the ‘Public transport travel times’ and ‘Routing parameters’ subsections.

The cumulative accessibility can be verbally expressed as the total number of services or opportunities that can be reached within a given travel time. We also include a relative cumulative subtype measure. Here, the weight of opportunities is represented as *W*_*jk*_/∑_*jk*_*W*_*jk*_.

Meanwhile, if a service cannot be easily interchanged or is scarce, the dual or travel time to the nearest facility is estimated. Formally, this is given by the following formula using the same notation:

$${A}_{ik}=\underset{j=1}{\overset{n}{\min }}\{{c}_{ij}\,:\,{W}_{kj} > 0\}.$$

This can be verbally expressed as the travel time to the closest service, e.g. to the main urban centre or hospital.

### Origins

LSOAs in England & Wales and DZs in Scotland are Table 1 equivalent geographical area systems used for statistical purposes in GB^19^. Accessibility indices were estimated for all LSOA/DZs as defined for the 2011 census in GB. The total adds up to 41,729 units. Their mean population and surface area is 1,562 inhabitants and 5.56 Km^2^, respectively (Table 1). The specific location of an origin is represented by the corresponding population weighted centroid. For England and Wales, LSOA’s centroids were accessed from the UK Government open data portal (https://data.gov.uk/) on the 2021-12-12 (version last updated on 2019-12-21). DZ centroids are produced by the Scottish Government and were manually downloaded from the UK Government’s open data portal (https://data.gov.uk/) (version last updated on the 2021-03-26).

**Table 1:** Descriptive statistics of the origins.

LSOA/DZ	England	Scotland	Wales	Great Britain (Total)
Count (N)	32,844	6,976	1,909	41,729
Population 2020 (mean)	1,722	784	1,660	1,562
Surface area sq. km. (Mean)	4.05	11.17	11.12	5.56

The location-based accessibility measures included in the dataset, like any spatially aggregated measure, are susceptible to the Modifiable Areal Unit Problem (MAUP), stemming from the arbitrary spatial aggregation choices for individual events^20,21^. As suggested in the MAUP literature, this implies that large zones in rural regions, and generally areas with lower density, are likely to introduce additional measurement error and further internal heterogeneity of the population and the features represented by each unit. Therefore, users are advised to proceed with caution, particularly when making comparisons between urban and rural geographies.

### Key services at potential destinations

The weights at potential destinations in the accessibility indices are represented by eight key services, namely employment, GPs, hospitals, primary schools, secondary schools, main urban centres, subcentres, and supermarkets. Table 2 presents a summary of the services included in the dataset by constituent country. England concentrates the largest proportion for all the services, followed by Scotland, while Wales hosts the smallest proportion.

**Table 2:** Total key services at potential destinations.

Key service	England	Scotland	Wales	Great Britain (Total)
Employment (in millions)	26.30	2.48	1.29	30.07
GPs	6,560	922	405	7,887
Hospitals	1,174	246	90	1,510
Education: Primary schools	16,608	2,003	1,242	19,853
Education: Secondary schools	2,893	359	205	3,457
Urban centre: Main	146	23	13	182
Urban centre: Subcentre	336	50	35	421
Supermarkets	5,467	672	339	6,478

In this dataset, the location of a service is represented by the centroid of the LSOA/DZ in which it is located. This information is obtained directly from the source if available. Otherwise, a spatial join is performed overlapping the location of the service at the point level and the LSOA/DZ boundaries accessed from the UK Data Service Census Support boundary dataset (http://census.ukdataservice.ac.uk/get-data/boundary-data.aspx). When the location at the point level is not available, the post code is used to match the corresponding LSOA/DZ geography using the ONS Postcode Directory (ONSPD, edition August 2021, accessed online via the Open Geography Portal on https://geoportal.statistics.gov.uk/). This approach implies that the travel cost from an origin to a potential destination is effectively the travel time to the LSOA/DZ centroid that contains a service *k*. In the remainder of this descriptor, the location of a service is interpreted according to this procedure.

#### Employment

The employment figures are obtained from the Business Register and Employment Survey and were accessed via Nomis (https://www.nomisweb.co.uk/). This considers part-time or full-time job positions of all industries. Also, the figures include working owners. However, these do not consider voluntary workers, self-employed, and working owners who do not pay their taxes on a pay as you earn (PAYE) basis. The year of reference is 2020, as it was the latest available at the time of accessing the information. The figures are aggregated at the LSOA/DZ level from the source.

#### General medical practices (GPs)

Information about GP practices in England and Wales is published by the National Health Service (NHS) Digital web platform (https://digital.nhs.uk/services/organisation-data-service/export-data-files/csv-downloads/gp-and-gp-practice-related-data). Specifically, we used the ‘epraccur’ data, which lists GPs at the individual level and updates them quarterly. The date of reference is November 2021. The following filters were implemented:

- Status code is “Active”, and;
- The prescribing setting is “GP Practice” only. This excludes any other setting category, for example: “Other”, “WIC Practice”, “OOH Practice”, “Community health service”, “Police Custody”, or “Optometry Service”.

GPs information for Scotland comes from the Public Health Scotland platform organised in the ‘Practice details’ dataset (https://www.isdscotland.org/). The date of reference is October 2021, which was the closes available to the travel time estimates at the time of download. The GP include:

- Dispensing practices, and;
- Type of practice ‘17 C’, ‘17 J’, or ‘2 C’ (This classification defines the type of contract, e.g. run by NHS, and/or other partners additional information can be accessed at https://www.isdscotland.org/Health-Topics/General-Practice/Workforce-and-Practice-Populations/Glossary/).

All the listings are assumed to be active as this source does not contain a field specifying the status.

#### Hospitals

GB lacks a consistent or unique source listing or characterising hospital services. Thus, we build on previous work^18^ to include relevant establishments. Here, the criteria employed are designed to exclude services not handling emergencies or accepting general patients. This aligns the definition of accessibility measures with the inclusion of ‘valuable destinations’, in this instance, for the general population. Thus, the establishments including the following key words were excluded:

- Specialist hospitals, key words: ‘birth’, ‘maternity’, ‘eye’, ‘rheumatic’, ‘throat’, ‘nose’, ‘ear’, ‘neurology’, ‘neurosurgery’, ‘specialist emergency care’, ‘orthopaedic’, ‘heart hospital’, ‘children’ or ‘dental’;
- Mental health, psychiatric, learning disability, or elder care hospitals, key words: ‘mental’, ‘psychiatry’, ‘psychiatric’, ‘elder,’ elderly’, ‘learning disability’, ‘learning disabilities’, or ‘psychogeriatric’;
- Day hospitals, key words: ‘day hospital’ or ‘day care’;
- Committee, key word: ‘Committee’

The list of raw establishments for England comes from the NHS UK website available on the 6^th^ of January 2022 (https://www.nhs.uk/about-us/nhs-website-datasets/). Additionally, establishments of the sub-type ‘Mental Health Hospital’ were excluded.

Data for Scottish establishments were obtained from Public Health Scotland (https://www.opendata.nhs.scot/) using the ‘Hospital Codes’ file for the date of reference January 2022.

The data used for Wales was assembled from a hospital directory published by the Health in Wales website (http://www.wales.nhs.uk/). The information was consulted in January 2022. An initial cleansing was carried out based on the classification offered by the source, where the following type of services were excluded: ‘CHC Local Committee’, ‘Psychiatric’, ‘Elderly Mental’, or ‘Day Hospital’. The key-word filtering process outlined before was also implemented afterwards.

#### Education: Primary schools and secondary schools

The location of education establishments in England comes from the Department for Education (DfE). The ‘All establishment data’ list was accessed via the Get Information about Schools (GIAS) platform (https://get-information-schools.service.gov.uk/) for the date of reference November 2021. The filtering criteria follow previous work, which includes the following establishments:

- Open establishments (at the date of reference);
- The admission policy is not ‘Selective’;
- The type of establishment is one of the following:
  - ‘Community school’,
  - ‘Voluntary aided school’,
  - ‘Voluntary controlled school’,
  - ‘Foundation school’,
  - ‘Academy sponsor led’,
  - ‘Academy converter’,
  - ‘Free schools’,
  - ‘University technical college’,
  - ‘Studio schools’.

A primary school was defined if the phase of education is ‘Primary’, ‘Middle deemed primary’, or ‘All-through’. A secondary school is defined when it meets the following conditions:

- The phase of education is ‘Secondary’, ‘Middle deemed secondary’, or ‘All-through’;
- The statutory low age is less than 16, and;
- The statutory high age is greater or equal to 16.

The source of information of education establishments in Scotland is the school education statistics collection published by Scottish Government (https://www.gov.scot/). The data employed is the ‘School contact details’ for the date of reference 13th of September 2021 (https://www.gov.scot/publications/school-contact-details/). The primary/secondary classification of schools used was the one included in the original source.

Welsh schools’ locations were accessed from the ‘Address list of schools in Wales’ published by the Welsh Government services and information website (https://gov.wales/address-list-schools#description-block). The date of reference is January 2022. A school was considered as primary school if the sector category was ‘Primary’ or ‘Middle’. A school was deemed as secondary if the sector label was ‘Secondary’ or ‘Middle’.

The educational establishments considered for this dataset do not include independent (private) schools because it is assumed they are not options available to all the population.

#### Supermarkets

The source of information used to consider the location of supermarkets is OpenStreetMap (OSM, www.openstreetmap.org). This choice is aided by the lack of official open-access data covering the whole GB. The OSM data was retrieved through an overpass API on February 2022 using the ‘osmdata’ package^22^ for R.

The feature defining the OSM query uses the following specifications:

- Bounding box/area corresponds to the whole UK,
- *key* is equal to ‘shop’,
- *value* is equal to ‘supermarket’.

The raw results were transformed into vector data as point or polygon geometries (multipolygons were discarded). The point elements were included if the name of the establishment was not missing. Records that included polygon geometries were included if the floor area was larger than 280 m^2^, according to the Sunday Trading Act 1994^23^. Later, the geometric centroid was computed and combined with records including point information. The following matches were implemented to refine the records:

- The amenity field is not missing,
- The brand or name field matches one of the following key words:

- ‘Lidl’,
- ‘ALDI’,
- ‘Co-op’,
- ‘Sainsbury’,
- ‘Tesco’,
- ‘Asda’,
- ‘Morrisons’

Lastly, only elements within GB were kept.

#### Urban centres

The definition and location of main urban centres and subcentres adopts the method proposed in Zhang and Pryce^24^. This draws on information from OSM and functional economic regions given by travel to work areas (TTWA) as defined for the 2011 Census (https://data.gov.uk/dataset/30aced27-4b4e-4f28-a14b-e982a0fccb65/travel-to-work-areas-2011). The method identifies a main urban centre for each TTWA in GB and a set of sub-centres based on their population.

### Public transport travel times

The public transport travel time between origins and potential destinations is estimated using the ‘r5r’ software version ‘0.6.0’ for R^25^. This package draws on R5, a multimodal open-source routing engine which allows the computation of detailed door-to-door routes^16,26^. The estimates use the road and pedestrian network and public transport schedules as primary inputs. The source of the former is OpenStreetMap (OSM), which was manually downloaded in PBF format from the Geofabric web platform on the 2021-11-22 for Great Britain. The public transport timetables were obtained from the following sources:

- Bus Open Data Service (BODS, https://www.gov.uk/transport/bus-services-routes-and-timetables), which includes information related to bus, tram, and ferry services. The data was downloaded in GTFS format on the 2022-11-22 from the ‘All - Download timetables data in GTFS format’ option which includes the timetables for the whole of GB, and;
- Rail Delivery Group, which primarily includes data on national rail passenger train services or heavy rail (https://www.raildeliverygroup.com/). This was manually downloaded on the 2022-11-22 in Common Interface File (CIF) format. The latter data was converted from CIF to GTFS format using the UK2GTFS package for R^27^.

The travel time was estimated from each origin to every potential destination (represented by the LSOA/DZ population weighted centroid), constituting a travel time matrix, according to the parameters described below.

### Routing parameters

The routing parameters used are adapted from Journey Time Statistics (JTS)^18^ where possible. This decision is aimed at maintaining as much consistency and comparability as possible with previous work. It should be noted that it is not possible to specify all of the model’s parameters equally since the routing software and data sources used for this work and the JTS are different. The specific criteria adopted in the present work is detailed below.

The journey routes were calculated departing on Tuesday November 22^nd^, 2021, within a three-hour time window from 7 a.m. to 10 a.m., representing a typical business day unaffected by major national holidays. This time and date choice is designed to represent a general accessibility measure, reflecting the period of highest demand for the transport system. For instance, in England, The number of trips in progress between 8 a.m. and 9 a.m. are three times higher than the average number of trips per hour^28^. Thus, this selection offers insights into the public transport capacity and effectiveness of the service at peak times when it is required the most. However, this approach does not capture the variability of public transport services at off-peak times. For specialised applications, such as the study of the nighttime economy, alternative times or departure dates should be considered.

The travel time estimates consider a combination of walking and all public transport modes included in the timetables described earlier (i.e. bus, tram, light rail transit, ferry, and heavy rail. Note that inter-city coaches are not included due to the incompatibility of the format of data with the routing software used). The approach adopted is the door-to-door. This considers the time needed for the following stages in a journey: walking from the specific origin to a public transport station (access time), on-board duration on a public transport vehicle, transfer and on-board duration on the next vehicle, and walking from the last station to the final destination. Also, the estimates include the initial waiting time for boarding a public transport vehicle. This time varies according to the selected time of departure and the timetables of the services required for a journey.

The variability of public transport journeys’ travel time estimates is captured by defining a time-window. The routing software considers a set of feasible travel time durations either by estimating several journeys using distinct times of departure within the travel time window or implementing Bootstrap techniques if the public transport services are defined in a ‘frequency’ file in the GTFS data^26,29^. A representative travel time is returned in the form of percentiles from the distribution estimated. Percentiles can be interpreted in terms of flexibility of travellers to adjust their time of departure in order to minimize their waiting and overall travel time. A low time percentile implies that the users are flexible to adapt their departure time according to the timetable whilst a high percentile is appropriate to represent users who do not rely on the schedules, e.g. in local journeys where the traveller expects to show-up and ride. While we produced the information for the 25th, 50th, and 75th percentile with the aim to provide more comprehensive information for the final users, we used the 50th percentile (i.e. the median) for the computation of accessibility indices.

Considering the above, the parameters employed for the computation of public transport journeys are the following:

- The time of departure considers a three-hour time window (180 minutes) which covers the same period of time as previous work (JTS), i.e. from 7 a.m. to 10 am. This is intended to capture the conditions at the morning peak for public transport routes.
- The maximum travel time allowed is limited to 120 minutes, regardless of the distance travelled.
- The maximum number of in-vehicle rides is set to three.
- The walking distance to egress/access public transport is not limited as long as the maximum travel time journey is not exceeded (120 minutes). This criterion implies that some of the routes can be completed by walking only if there is not a public transport service available or that enables an earlier time of arrival. Walking-only routes represent approximately 0.3% of the OD routes estimated.
- The walking speed is set to 4.8 Kph (following the on road/path network used in the JTS).

The dataset considers walking-only journeys given that literature regards walking and public transport as complementary and integrated modes of transport^30,31^. Overlooking these options lead to the formulation of unreasonable routes, potentially yielding longer travel times compared to walking only.

## Data Records

The dataset is available from the Urban Big Data Centre Data hub (https://data.ubdc.ac.uk/dataset/public-transport-accessibility-indicators-2022) and a long-term Zenodo repository^32^. The dataset was accompanied by a technical note which was made available at the time of publication^33^. The records of this dataset are compiled in two main products. The first contains the raw travel time estimates represented in a TTM. The second, includes the public transport accessibility indicators to different services.

### Travel time matrix

The TTM can be used as the main input to tailor or develop further accessibility indicators according to the specific needs of users. Additionally, it could be a useful input for other analyses, such as spatial network analyses. Table 3 presents a description of the contents of the ‘ttm/pt/descrip_ttm_pt.csv‘ file which includes the all-to-all TTM.

**Table 3:** Contents of the travel time records.

Variable	Type	Description
fromId	nominal	2011 LSOA/DZ geo-code of origin
toId	nominal	2011 LSOA/DZ geo-code of destination
travel_time_p025	numeric	25 travel time percentile by public transport in minutes
travel_time_p050	numeric	50 travel time percentile by public transport in minutes
travel_time_p075	numeric	75 travel time percentile by public transport in minutes

### Accessibility indicators

Table 4 and Table 5 describe the accessibility indicators generated for each of the key services included in the present data collection. It includes: (1) the directory of the output file; (2) the accessibility measure computed for the respective service, namely cumulative, relative cumulative, and/or minimum travel time, and; (3) a description of the measure computed.

**Table 4:** Summary of the public transport accessibility indicators records.

File	Measure	Description
*Employment*		
employment/ access_employment_pt.csv	Cumulative: time cut 15, 30, 45, 60, 75, 90, 105, 120	Number of employment positions within N minutes by public transport
employment/ access_employment_pt.csv	Relative cumulative: time cut 15, 30, 45, 60, 75, 90, 105, 120	Number of employment positions within N minutes by public transport divided by the total positions in Great Britain
*GPs*		
gp/access_gp_pt.csv	Cumulative: time cut 15, 30, 45, 60, 75, 90, 105, 120	Number of GPs within N minutes by public transport
gp/access_gp_pt.csv	Relative cumulative: time cut 15, 30, 45, 60, 75, 90, 105, 120	Number of GPs within N minutes by public transport divided by the total number of GPs in Great Britain
gp/access_gp_pt.csv	Minimum travel time	Travel time in minutes to the closest GP
*Hospitals*		
hospitals/access_hospital_pt.csv	Cumulative: time cut 15, 30, 45, 60, 75, 90, 105, 120	Number of hospitals within N minutes by public transport
hospitals/access_hospital_pt.csv	Relative cumulative: time cut 15, 30, 45, 60, 75, 90, 105, 120	Number of hospitals within N minutes by public transport divided by the total number hospitals in Great Britain
hospitals/access_hospital_pt.csv	Minimum travel time	Travel time in minutes to the closest hospital

**Table 5:** Summary of the public transport accessibility indicators records, continued.

File	Measure	Description
*Education: Primary schools and secondary schools*		
schools/access_school_pt.csv	Cumulative: time cut 15, 30, 45, 60, 75, 90, 105, 120	Number of primary schools within N minutes by public transport
schools/access_school_pt.csv	Relative cumulative: time cut 15, 30, 45, 60, 75, 90, 105, 120	Number of primary schools within N minutes by public transport divided by the total primary schools in Great Britain
schools/access_school_pt.csv	Minimum travel time	Travel time in minutes to the closest primary school
schools/access_school_pt.csv	Cumulative: time cut 15, 30, 45, 60, 75, 90, 105, 120	Number of secondary schools within N minutes by public transport
schools/access_school_pt.csv	Relative cumulative: time cut 15, 30, 45, 60, 75, 90, 105, 120	Number of secondary schools within N minutes by public transport divided by the total secondary schools in Great Britain
schools/access_school_pt.csv	Minimum travel time	Travel time in minutes to the closest secondary school
*Urban centre; city and greater city*		
urban_centre/access_cities_pt.csv	Minimum travel time	Travel time in minutes to the closest city
urban_centre/access_cities_pt.csv	Minimum travel time	Travel time in minutes to the closes greater city
*Supermarkets*		
supermarket/ access_supermaet_pt.csv	Cumulative: time cut 15, 30, 45, 60, 75, 90, 105, 120	Number of supermarkets within N minutes by public transport
supermarket/ access_supermaet_pt.csv	Relative cumulative: time cut 15, 30, 45, 60, 75, 90, 105, 120	Number of supermarkets within N minutes by public transport divided by the total number of supermarkets in Great Britain
supermarket/ access_supermaet_pt.csv	Minimum travel time	Travel time in minutes to the closes supermarket

**Fig. 1:**
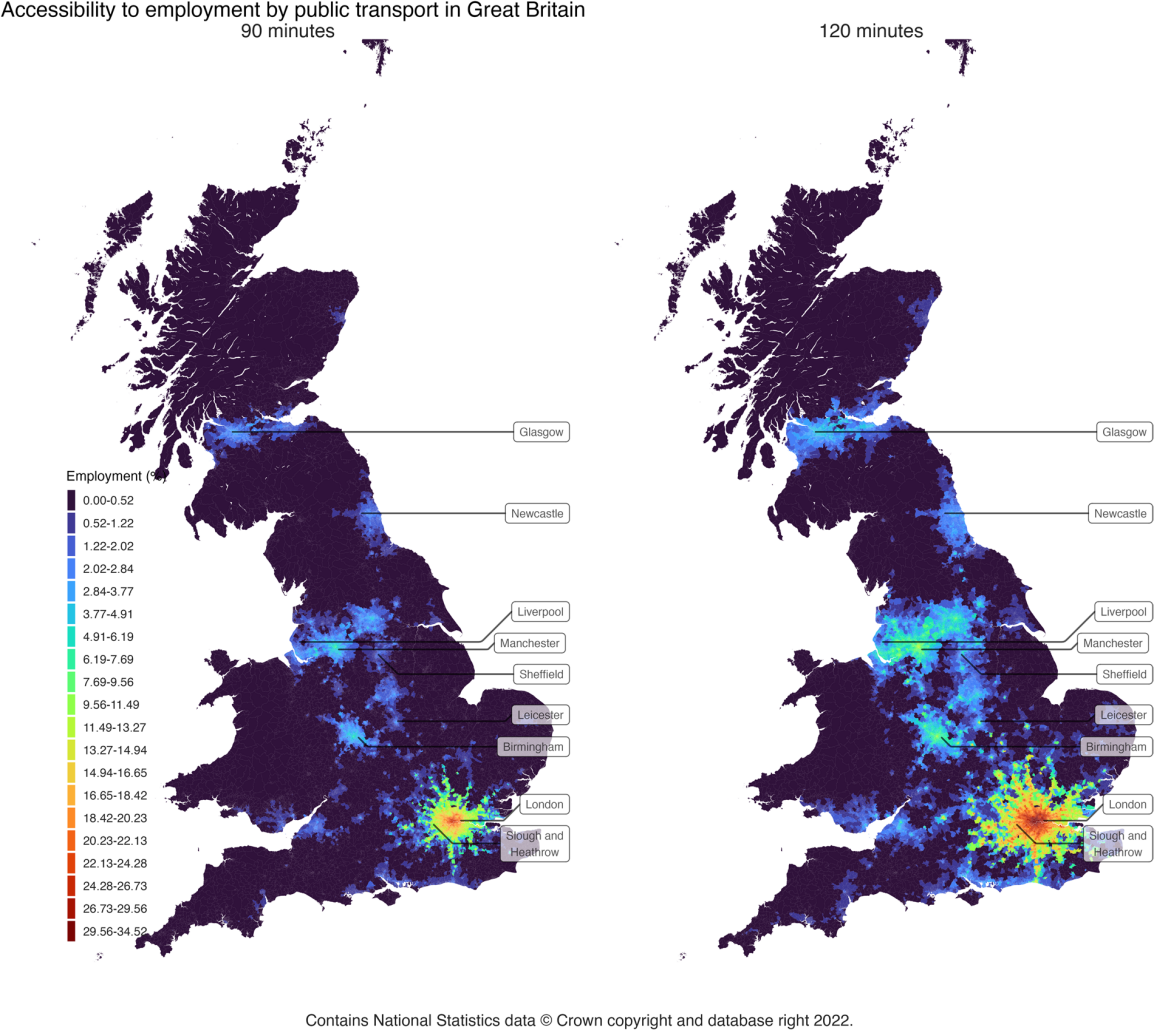
Visualization of public transport accessibility indicator to employment as percent of the total for 90 minutes and 120 minutes.

Figure 1 visualizes the relative cumulative accessibility indicator to employment for the whole GB for two time-thresholds as an illustrative example. The indicator is shown as the percentage of the total employment that can be reached within 90 minutes and 120 minutes from each LSOA/DZ, left and right respectively. In both examples, London plays a dominant role. Other clusters, such as the corridor formed between Liverpool, Manchester and Leeds, cover relevance at the regional level if a 120-minutes threshold is considered. Birmingham is also highlighted as being well-positioned. This is possible because additional employment located in London is also considered as available from further locations.

The files ending in ‘_car.csv‘ contain similar accessibility indices comparable to those previously described but are calculated by car. These employ estimated travel times derived from a free-flow traffic model, and have not undergone validation. These limitations should be acknowledged when using these measures.

## Technical Validation

### Travel time estimates

The travel time estimates were validated by comparing it with two external data sources, namely mobile phone app location data and the Distance Matrix API of the Google Maps platform (Google). During the assessment process, it is important to consider that “there is no absolute and ‘correct’ travel time between locations, as the routes and their conditions are always generalisations of reality”^34^. Several elements might affect the estimates generated by different tools or sources, such as the variability of public transport operation, individual physical and psychological characteristics, knowledge of timetables, and randomness. Part of this is captured in our estimates, as discussed in the Methods section.

First, we validate the travel time estimates generated by r5r using app location records from the GlaMAS dataset held by the Urban Big Data Centre. As part of the data collection, around 400 people in Glasgow, Scotland, installed the MyWays app on their smartphone which tracked their movements between February and October 2022. Some users ran the app for a few days, while others run it for a few months. The raw movement data was processed by the app provider, TravelAI. They classify routes which connect stops e.g., travelling from home to work is a route. Each route is made up of one or more leg. Each leg has one mode of transport. We select all routes where a mode of public transport has been detected for at least one leg. We exclude any routes where a car is used for any leg, because we have run r5r without allowing the usage of a car. This process gives a table of routes with the latitude and longitudes of the origins and destinations. We also know the date and time when the trip started and the duration of the trip. We use the origin, destination and start time to predict a journey time using r5r. This is then compared.

It is worth noting that the GlaMAS app data has some limitations. Stops, moves, and travel modes are automatically detected, and this process is subject to error. To mitigate this, routes with an average speed lower than 4.8 Kph were excluded based on the notion this is the lowest possible traveling speed. In addition, we do not have access to the public transport timetables for the exact dates the people travelled on. Still, in the absence of ground-truth data, this dataset allows us to compare our modelled journey time. Additionally, we only have this kind of data for Glasgow city-region.

**Fig. 2:**
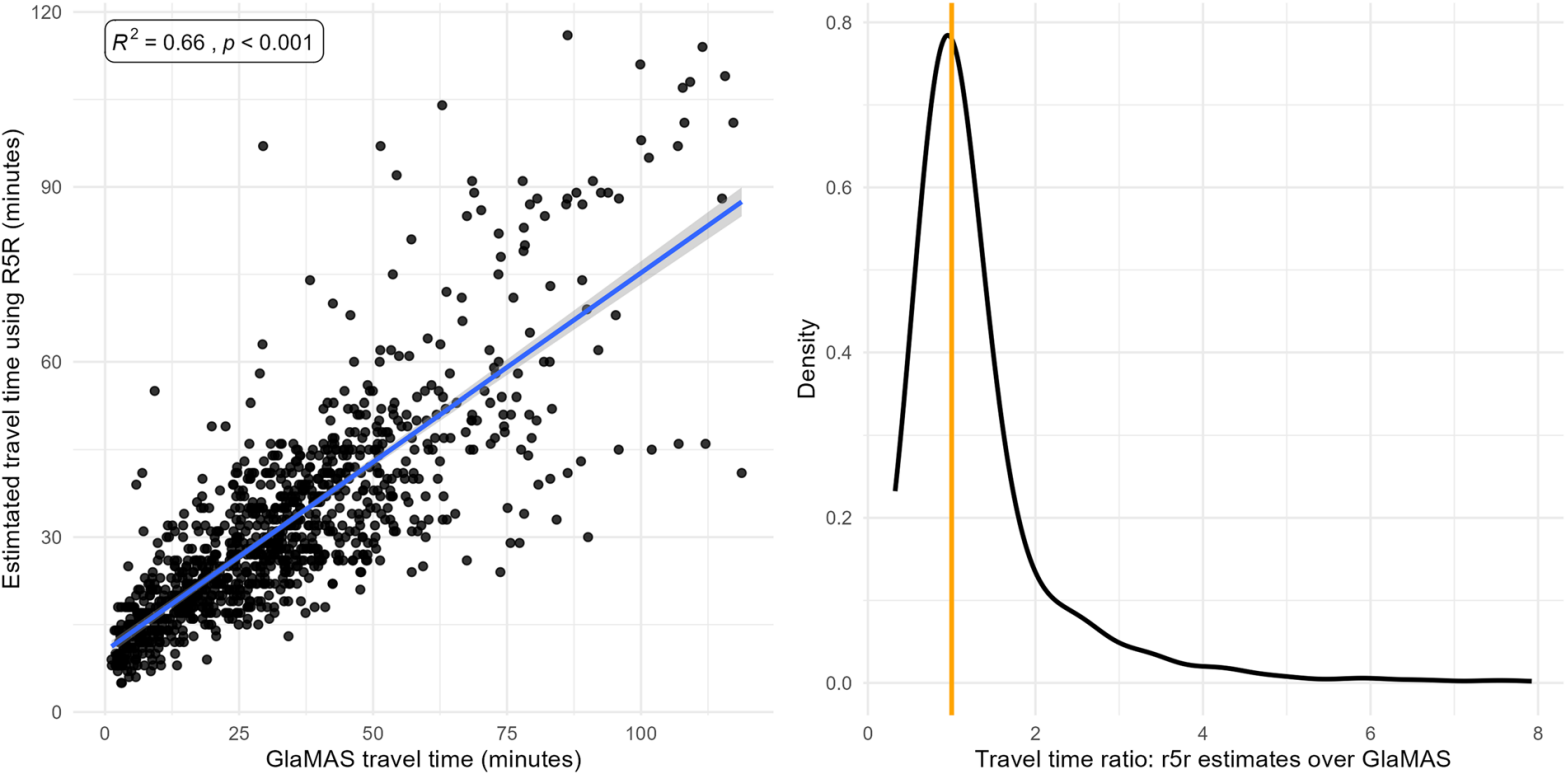
Travel time comparison between r5r estimates and GlaMAS travel time. Panel a), on the left, shows the scatter plot with a positive correlation. Panel b), on the right, shows the distribution of the ratio difference of r5r estimates over GlaMAS.

We compared a sample of 1,089 journeys. The mean travel time for both sources is very similar, at 32.8 minutes for the GlaMAS routes versus 31.3 minutes for the r5r estimates. The standard deviation is 23 and 17.6, respectively. This aligns with expectations, as there is additional noise in the tracked travel times. The results of the comparison are visualized in Fig. 2. Panel a), on the left, shows a strong positive correlation (R² = 0.66) with higher uncertainty for trips over one hour. Panel b), on the right, illustrates the distribution of the ratio of the r5r estimates to GlaMAS travel times. The mean ratio difference is 1.32, implying that, on average, r5r estimates are longer than those in the GlaMAS dataset. The interquartile range is 0.87 to 1.42, reflecting good correspondence. Overall, the sources compared are consistent.

Second, we compared our travel time estimates at the national level with Google estimates, which has been deemed a reliable source in various studies^34,35^. The primary objective is to validate the estimates produced by our routing model in terms of assumptions, routing parameters, and the road and pedestrian network used. A potential limitation is that Google may utilize similar public transport timetables as its main input. However, this is uncertain, as their methodology is not openly disclosed. Therefore, this comparison does not evaluate the quality of the timetable data, a concern addressed in the previous validation stage.

**Fig. 3:**
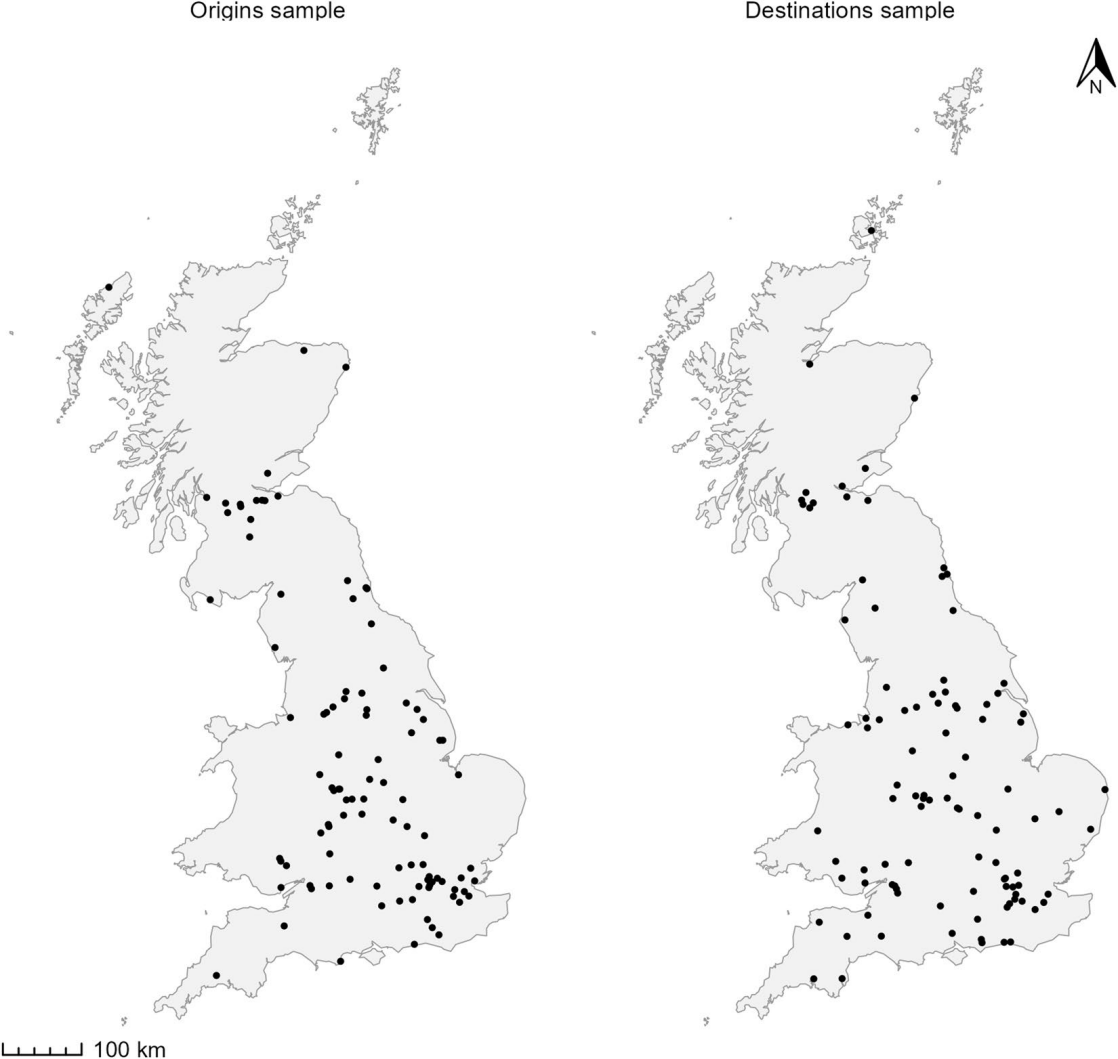
Selected random origin-destination samples used to validate the travel time estimates by public transport in Great Britain.

We selected a random sample of 100 LSOA/DZ centroids representing origins and another 100 representing destinations. Figure 3 shows the location of the randomly selected points constituting the samples for the second validation process.

We estimated the travel time from each origin to every potential destination in the samples using both our local routing engine (R5R) and the Google Distance Matrix API. The date and time of departure for the Google estimates was Tuesday 11^th^ of January 2022 at 07:00 a.m. This differs from the date specified for our estimates because Google does not allow requesting past dates. However, we selected a representative business day which would be comparable to our estimates. The mode selected was ‘transit’ and the rest of the parameters are given by default. From the possible OD combinations, we only considered OD pairs in which the origin and the destination was different. Also, the maximum travel time considered in both estimates was 150 minutes, allowing a 30-minutes tolerance in addition to the 120 minutes threshold used for the TTM. This is to allow a comparison for trips that otherwise would not be included if one of the sources exceeded the initial duration limit established.

**Fig. 4:**
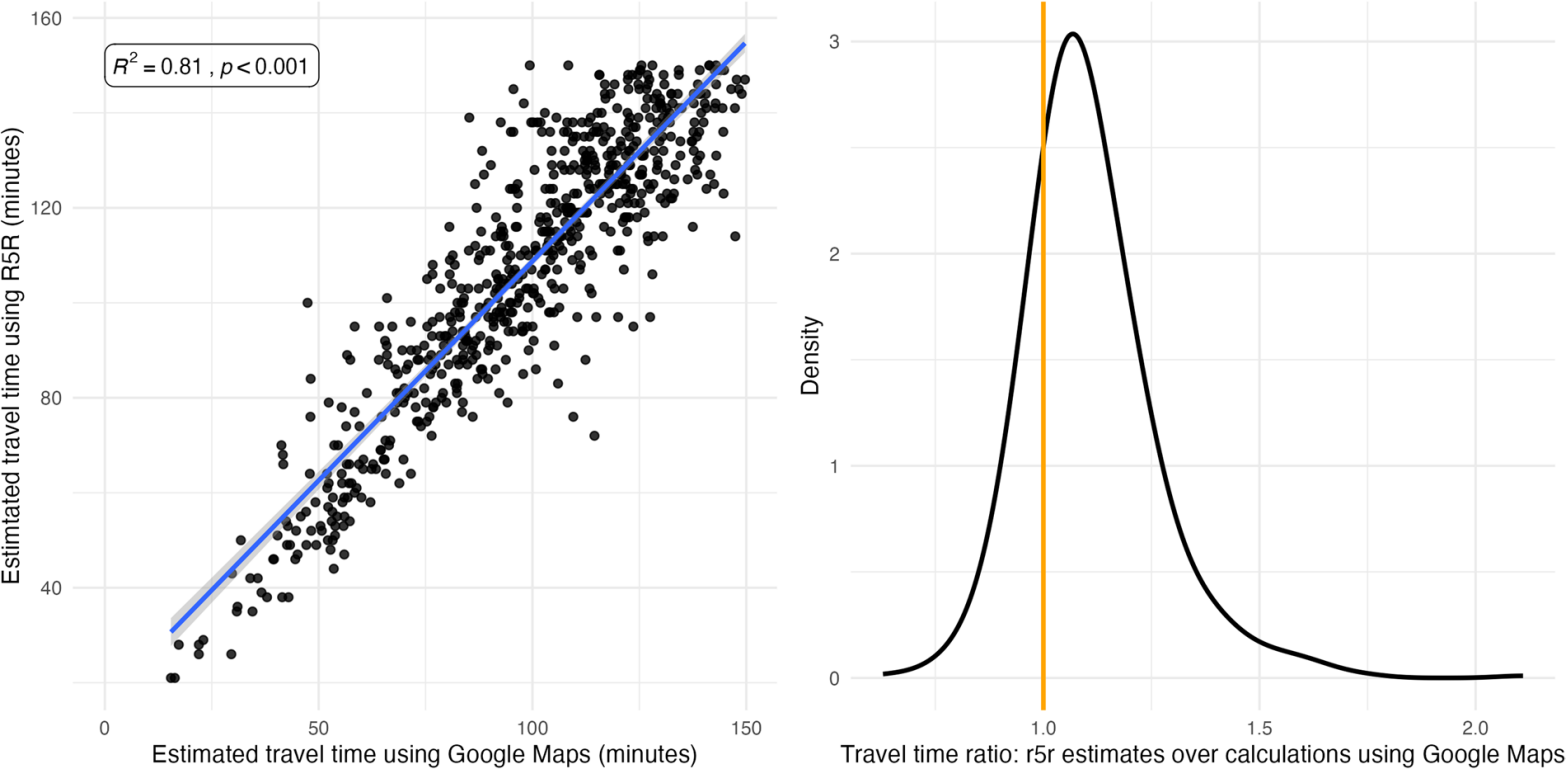
Travel time estimates comparison of our 50^th^ quantile estimates and using Google Maps of a randomly selected origin-destination pairs sample (N = 677).

Panel a) on the left of Fig. 4 illustrates the relationship between our 50^th^ quantile travel time estimates and Google. The plot shows good consistency and high correlation between both sources. Panel b), on the right, shows the distribution of the ratio of our estimates over the calculations using Google, where 1 would implies identical results. It is shown that our estimates are slightly larger than Google, as the median is 1.08. Yet, 95% percent of the estimates fall between 0.91 and 1.38 of the ratio difference. The differences observed could result from the notion that Google estimates tend to minimise the initial wating time by suggesting an alternative time of departure from the specified. Meanwhile, our estimates consider this part of the trip according to the time-window approach discussed in the ‘Travel time matrix’ subsection of the Methods section.

### Location of services

The locations of most identified destinations are derived from official sources, which are generally deemed trustworthy considering the dedicated resources to collect the information, transparency in the processes, accountability, and public trust. Users seeking further details are encouraged to consult the relevant sources. The location of urban centres uses both official as well as crowdsourced data and follows a method reported in a scientific publication which provides explicit details about the rationale and supporting evidence^24^.

In the absence of official open-access data to identify the location of supermarkets, we opted for crowdsourced data, i.e. OSM. We cross-validated the completeness of the OSM data using a commercial dataset provided by the national mapping agency for Great Britain, the Ordnance Survey (OS). The ‘Points of Interest’ dataset was accessed through the Digimap service operated by EDINA^36^. We found that the supermarket locations used for the accessibility indices represent about 80% of the total reported in the OS data. This may arise from the completeness and potential biases present in the OSM data. Additionally, the update date for the OSM data is February 2022, while the OS data is updated for June 2023. This can introduce additional discrepancies as some locations may have opened after the date recorded in OSM.

This limitation represents minor implications for the dual or closest supermarket measure because this does not consider the size of the service. For instance, If OSM reported one establishment and OS records three, then, it would not make a substantial difference as only the travel time in minutes to the closest service is reported. However, cumulative measures are expected to be more affected. Overall, accessibility to supermarkets would be underestimated. This is more meaningful for absolute cumulative measures than for relative cumulative ones.

## Usage Notes

As briefly mentioned in ‘Background & Summary’ section, this dataset can be used at various levels, namely at a foundational, integrative, and advanced or strategic level. At the foundational, any of the readily available accessibility indices can be directly included into the analysis at a small statistical official unit by joining it to other dataset using the unique LSOA/DZ code. This can be valuable for transportation and planning agencies to conduct performance management analyses. This offers the advantage of considering both land use and transportation developments. Public health bodies, for example, can also use the indices to support their service offer plans. At this level, the users can choose from a wide variety of services and time-cuts according to previous knowledge or specific needs.

As mentioned in the ‘Background & Summary’ section, location-based measures are valuable equity indices. For distributional analysis, the health, employment, or education indices provided can be coupled to official population stratifications available for all regions in GB. Some examples are the Scottish index of multiple deprivation (https://www.gov.scot/collections/scottish-index-of-multiple-deprivation-2020/) or English Indices of deprivation (https://www.gov.uk/government/collections/english-indices-of-deprivation). As these indices are also estimated at the same geographic level (LSOA or DZ), it is possible to directly use the ‘geo_code’ column with the unique LSOA/DZ code included in such datasets to combine the information. This can be done using a variety of statistical software, e.g. R programming language, SPSS, or Office Excel.

A primary form of usage commonly involves the spatial visualisations. The ‘InFuse’ 2011 geographies for GB dataset contains the boundaries associated to each LSOA/DZs. This is openly available from the UK Data Service platform (https://infuse.ukdataservice.ac.uk/help/definitions/2011geographies/index.html). The geographies can be matched with the accessibility indices using the ‘geo_code’ column which is present in both datasets. The manipulation and operation can be achieved using conventional geographic information (GIS) software, e.g. QGIS, ArcMap, or packages for spatial data manipulation for R or Python programming languages, e.g. SF or Arcpy, respectively.

### An integrative usage illustrated by the 20-minute city

At an integrative level, a user can tailor or derive further indices by combing or aggregating the information included in this dataset geographically or thematically. An intermediately-engaged user can aggregate the indices thematically by identifying locations that have a hospital and a GP available at given threshold, for example. This could aid the planning process of facilities addressing the needs of population groups that more frequently require health services, e.g. retirement homes, or caring centres for people with additional support needs. Also, the TTM can be used to estimate the accessibility level of services not included in this data set, or where the use of a different impedance function is needed, e.g. as in gravity-based accessibility measures.

A specific use case of this type of usage can be illustrated drawing on the ‘20-minute city’ concept. This has been defined as “a city that enables residents to access most of the activities needed for good living within a 20-minute walk, cycle or public transport trip from their homes”^37^. This notion has gained traction in both planning policy and the research agenda, and thus, this dataset can support further analyses. We focus on the commonly referred services or activities suggested in literature, namely education, healthcare, and food-related services^38^. The dataset allows to easily assemble a 20-minute score as similarly done in previous work for one UK city^38^. This was constructed by adding a point for each of the following services available within 20 minutes for each LSOA/DZ in GB: GP, hospital, primary school, secondary school, large urban centre, subcentre, and supermarket. We also leave out the measures directly related to employment. This results in a 0 to 7 score. The scale implies that values at or near 0 reflect a low number and diversity of key services available within 20 minutes, while values at or close to 7 denote the opposite. All the data management and processing were perform using R programming language (code available in the GitHub repository).

**Fig. 5:**
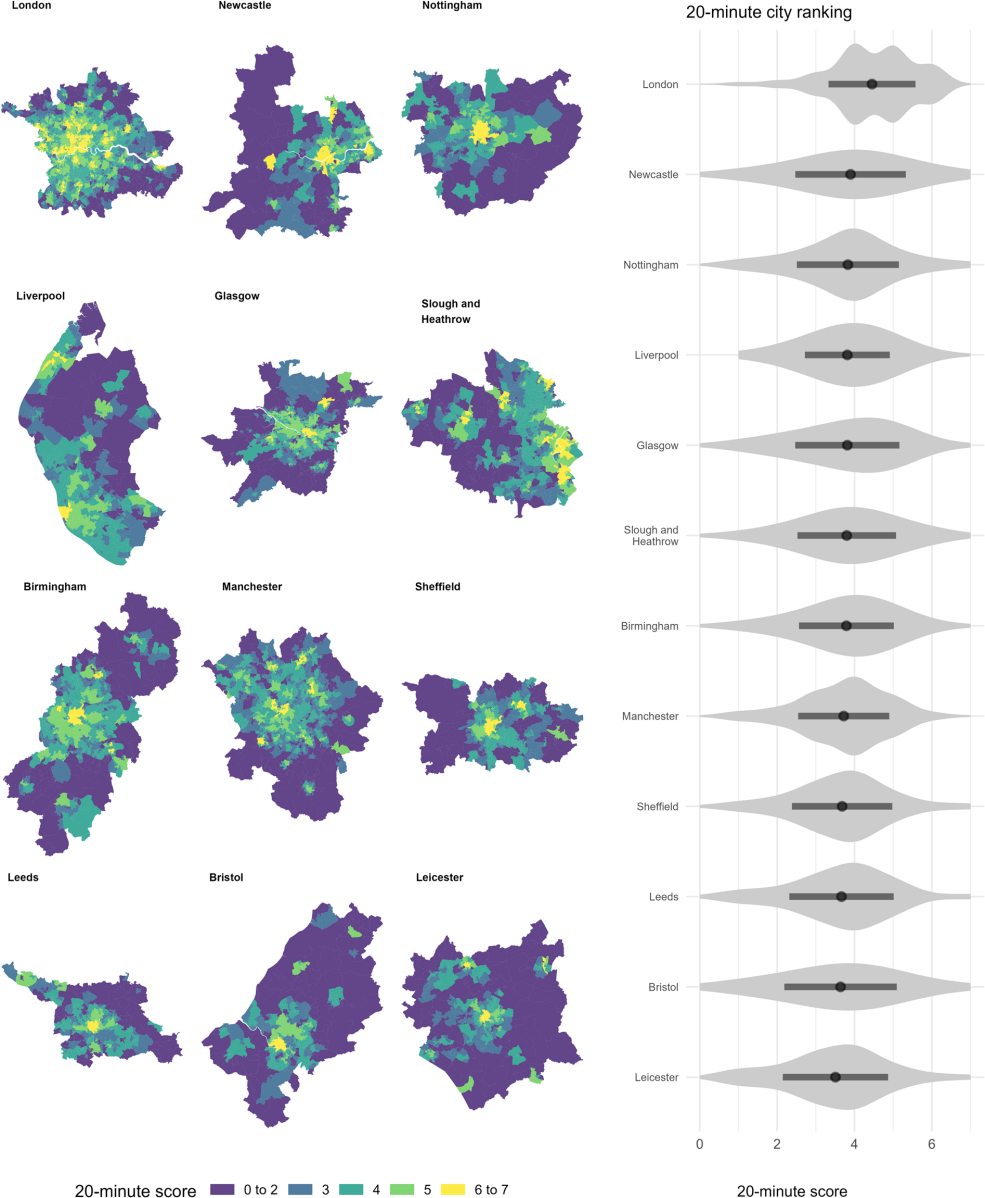
20-minute score for selected cities in Great Britain.

The 20-minute scores were grouped according to a set of selected cities in GB. The geographic definition of the cities was obtained from the 2011 Travel to Work Areas (TTWA) dataset (accessed from the ONS Open Geography Portal at https://geoportal.statistics.gov.uk/). We selected the 12 most populated TTWs for this illustrative example. The score was associated with a city by spatially joining the LSOA/DZs population weighted centroids using the ‘st_join()’ function of the SF package^39^ for R. Figure 5 illustrates the derived scores for the selected cities in GB. The left-hand side panel shows a series of mini-maps coloured by their corresponding score and ordered by the mean (from the highest to lowest, from top to right). The right-hand side panel shows the distribution of the score in the same order (from top to bottom). London resembles a polycentric-like structure which is both reflected in the map and the distribution of the score. Manchester depicts a similar pattern, but it is less apparent than the capital city. The rest of the maps also reveal further structural characteristics. For instance, Newcastle, Glasgow, Leeds, and Bristol, tend to present higher scores towards the north of their respective central cores.

The shape of the distribution also gives some relevant information. For instance, scores in Liverpool or Manchester are more concentrated around the mean with relatively short tails to both sides of the curve, implying that access to key services is more evenly distributed in the city with a few locations experiencing extreme low or high access. By contrast, Bristol or Newcastle, for example, show a flatter shape with long tails, suggesting higher levels of inequality. Specifically, while some residents can reach a high number and more diverse services (close to 7), a similar number experience very limited access to opportunities (close to 0).

### Further advanced or strategic usage

It is worth noting a deeper analysis which includes additional services in the 20-minute score is possible to derive from the products included in this dataset at an advanced or strategic level. This is because the TTM can allow the estimation of additional closest services if their location is available. This could directly use the TTM included in the dataset and supplemented with further external information. The procedure can be aided by the process followed for this work, as it is similar to the one shown in the code for the services included. This is also applicable to examining other specific services such as the accessibility to vaccination sites, as the COVID-19 uptake rate has been shown to be associated with the level of accessibility^6^.

Another form of usage would entail the reproduction of the indicators guided by the open-access code made available, the identification of data sources, and procedures followed using inputs corresponding to different time-periods or origin-destination geographies. This may be useful for updating the indicators or the development of ‘what-if’ scenarios in the British or broader international context. This would be facilitated by the open resources required, such as open-access data as the inputs, open-source software, and the good compatibility of inputs used for PT routing, i.e. standardised timetable and OSM data. Also, these can function as benchmark indices.

## Data Availability

All the source codes used for producing this dataset are openly available from the following link: https://github.com/urbanbigdatacentre/access_uk_open. The versions of the relevant software used are stated for each of the key elements in the main body of the paper.
